# Gastroduodenal artery aneurysm - an extremely rare but insidious cause of abdominal pain: a case report

**DOI:** 10.11604/pamj.2024.47.77.42605

**Published:** 2024-02-20

**Authors:** Dawood Shehzad, Dawlat Khan, Mustafa Shehzad, Wahab Khan, Hammad Chaudhry, Tony Oliver

**Affiliations:** 1 Department of Internal Medicine, Sanford School of Medicine, University of South Dakota, South Dakota, United States of America,; 2 Hackensack University Medical Center, Hackensack, United States of America

**Keywords:** Visceral aneurysms, gastroduodenal artery aneurysm, refractory abdominal pain, case report

## Abstract

An arterial aneurysm is a localized weakening of the artery wall that results in pathological dilatation. All intra-abdominal artery aneurysms are labeled as visceral artery aneurysms (VAA), apart from the aorto-iliac artery aneurysms. VAA´s are rare, gastroduodenal artery aneurysms (GDAA), constituting 1.5% of visceral artery aneurysms. A woman in her early 80s´ presented with chronic epigastric pain, weight loss, and nausea. Conservative management was unsuccessful. Imaging revealed a GDAA, prompting endovascular coil embolization. Subsequent evaluation confirmed Polyarteritis Nodosa (PAN), treated with rituximab. The report underscores the diagnostic challenges, emphasizing the need for a multidisciplinary approach using imaging and angiography. GDAA's potential life-threatening rupture necessitates prompt intervention, as illustrated in this case. The rare association with PAN, although infrequent, underscores the importance of considering underlying etiologies in multiple visceral aneurysms. Early diagnosis and intervention are pivotal for this uncommon yet potentially lethal condition.

## Introduction

All intra-abdominal aneurysms excluding aortoiliac axis aneurysms are referred to as visceral artery aneurysms (VAAs). It is an extremely rare entity, with a reported incidence of 0.01 to 0.2 % [1]. Gastroduodenal artery aneurysms (GDAA) are the rarest, compromising only 1.5 % of all VAAs [1] The most common clinical presentation is abdominal pain, less commonly it can present with nausea, vomiting, symptoms of gastric outlet obstruction, and jaundice. Rarely it can be an incidental finding. Atherosclerosis and chronic pancreatitis are among the major reported risk factors; however, liver cirrhosis, peptic ulcer disease, fibro-muscular dysplasia, and polyarteritis nodosa (PAN) have also been reported as risk factors in the literature [2,3]. Aneurysmal rupture can be a potentially fatal condition [3]. We report a case of a female in her early 80s who presented for 3 months of nonspecific epigastric pain, nausea, and vomiting ultimately found to have a gastroduodenal artery aneurysm.

## Patient and observation

**Patient information:** a female in her early 80s with a past medical history of essential hypertension presented for 3 months epigastric pain, nausea, non-bilious vomiting, reduced appetite, and seven pounds of unintentional weight loss. The pain was constant, dull, with no radiation, exacerbated by food intake, and not alleviated with oral antacid or proton pump inhibitors. There were no associated respiratory symptoms, skin rash, fever, hematemesis, melena or haematochezia haematuria, or numbness. She had three prior outpatient clinic visits for similar complaints and had no improvement with conservative management. She denied tobacco, ethanol, or illicit drug use.

**Clinical findings:** physical examination was remarkable for mild epigastric tenderness on superficial palpation, the rest of the systemic exam was unremarkable.

**Timeline of current episode:** symptoms started in August 2023, and had three outpatient visits between August 2023 and October 2023. Presented to the Emergency Department in November 2023.

**Figure 1:**
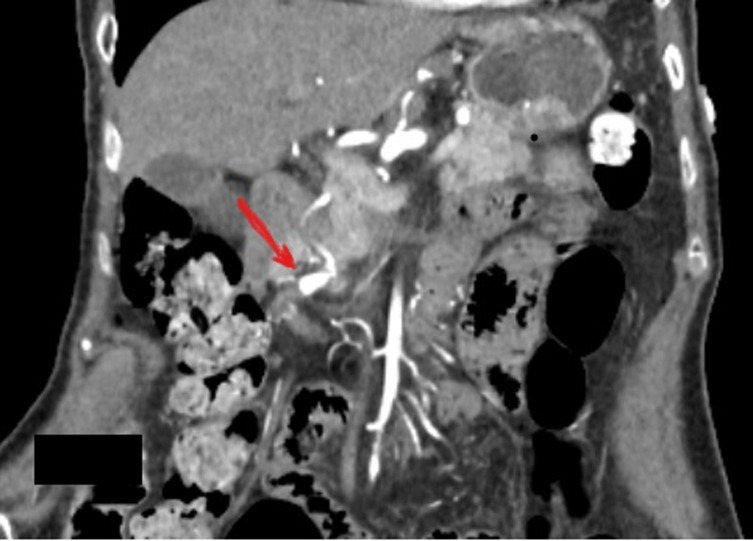
contrast enhanced computerized tomography of abdomen and pelvis-coronal sections showing gastro-duodenal artery aneurysm (arrow)

**Figure 2:**
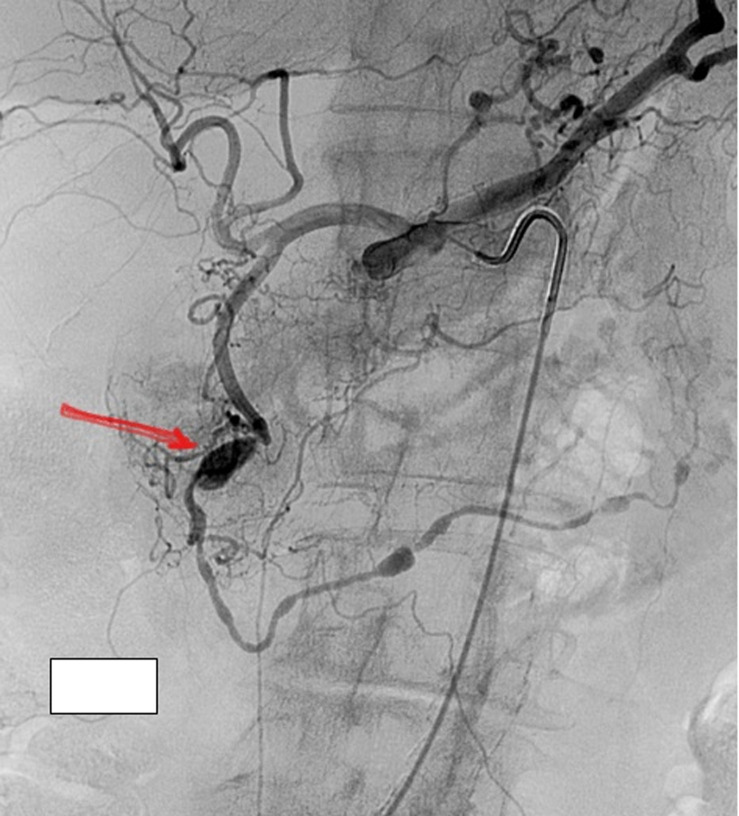
visceral angiogram showing gastroduodenal artery aneurysm (arrow)

**Figure 3:**
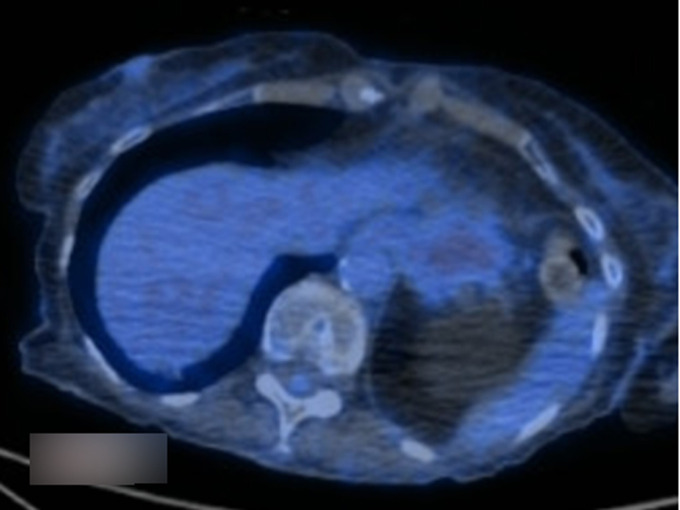
positron emission tomography (PET) scan and a computed tomography (CT) scan showing vasculitis of aorta

**Diagnostic assessment:** a complete blood count (CBC) revealed no anaemia, a normal white cell count, and platelets. Renal and liver function tests were also within normal limits. A C-reactive protein (CRP) was high, 22 mg/L (reference <5 mg/L). A hepatitis panel was also negative. Anti-neutrophil cytoplasmic autoantibody (ANCA) and antinuclear antibodies (ANA) that had been tested during one of her outpatient visits and were normal. An electrocardiogram revealed normal sinus rhythm with no ischemic changes. Given the chronicity of her symptoms, and failure of conservative management, she underwent an Upper Gastrointestinal (GI) endoscopy that showed mild gastritis, she was started on twice daily proton pump inhibitors (PPI) for four weeks. At four weeks, her symptoms still had not improved. A computerized tomography (CT) of the abdomen and pelvis with contrast revealed a GDA aneurysm and concerns for polyarteritis nodosa (Figure 1). A visceral angiogram showed a gastroduodenal artery aneurysm (Figure 2). A positron emission tomography (PET) CT scan revealed vasculitis of the bilateral internal carotids, subclavian, axillary, aorta, and iliac arteries (Figure 3).

**Figure 4:**
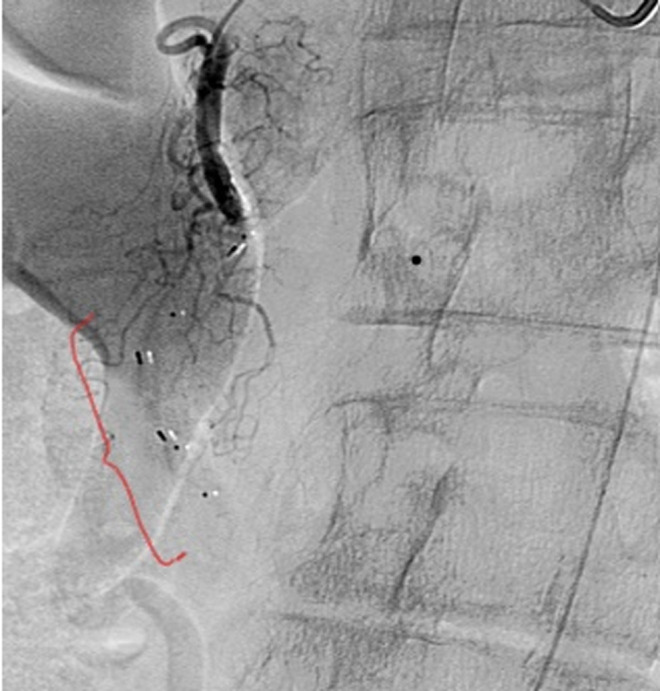
gastroduodenal artery (GDA) aneurysm post interventional embolization image, revealing multiple stents

**Therapeutic interventions:** a presumptive diagnosis of polyarteritis nodosa was made, and she was started on prednisone and later transitioned to rituximab. Her upper gastrointestinal symptoms persisted, and she underwent GDA embolization via a trans-femoral artery approach with stenting (Figure 4).

**Follow-up and outcome:** at 6 months after her procedure and starting of therapy, she reported complete resolution of symptoms. Her CRP had also normalized.

**Patient perspective:** the patient declined to give her perspective on her illness.

**Informed consent:** it was taken from the patient and is available with the authors in written form.

## Discussion

Gastroduodenal artery (GDA) aneurysm is an exceedingly uncommon yet a potentially deadly condition. A literature review revealed that only 74 cases were reported between 1956 and 2011 [1-3]. The overall rupture risk of VAA is 25%, whereas the rupture risk of GDA aneurysm is 75%, with up to 40% mortality, entailing a high suspicion level for diagnosis [1-3].

Visceral angiography has the highest sensitivity for diagnosis (100%), followed by CT abdomen with contrast (67% sensitivity), and US abdomen (only 50% sensitivity). Contrast-enhanced CT of the abdomen is non-invasive and broadly used [4,5]. Magnetic resonance imaging (MRI) particularly magnetic resonance angiography (MRA) is useful in cases where iodinated contrast would be limited like chronic kidney disease [6].

The presence of a gastroduodenal artery aneurysm necessitates treatment regardless of aneurysm size to lower the likelihood of rupture and related mortality [7]. Risk factors for rupture include pregnancy, pancreatitis, trauma, and aneurysm caused by surgical or endovascular intervention. Hemodynamic stability determines whether an open vascular surgical approach or an endovascular approach with coil embolization is used. Endovascular approaches are gaining popularity [7,8]. As our patient had mild symptoms, and was hemodynamically stable therefore she was able to undergo successful endovascular coil embolization of her lesion.

Polyarteritis nodosa (PAN) is a systemic necrotizing vasculitis that predominantly affects medium-sized muscular arteries, with small arteries also being involved. Unlike other vasculitides, it does not correlate with anti-neutrophil cytoplasmic antibodies [9]. PAN is an infrequent underlying aetiology of GDA aneurysms that should be assessed further, notably if numerous small visceral aneurysms are found in our patient [10]. It is treated with oral steroids and immunosuppressive medications (cyclophosphamide, azathioprine, and rituximab) depending on disease severity. Our patient had moderate-severe PAN, initially treated with high-dose steroids and later with rituximab resulting in complete symptom resolution.

## Conclusion

Gastroduodenal artery (GDA) is an extremely rare but sinister aetiology of abdominal pain that is associated with a high mortality rate if ruptured. Clinicians should be familiar with its diagnosis and treatment. Early interventional radiology or vascular intervention brings down mortality. Despite being an exceptionally rare cause of GDA, PAN has an excellent prognosis when caught in time.
